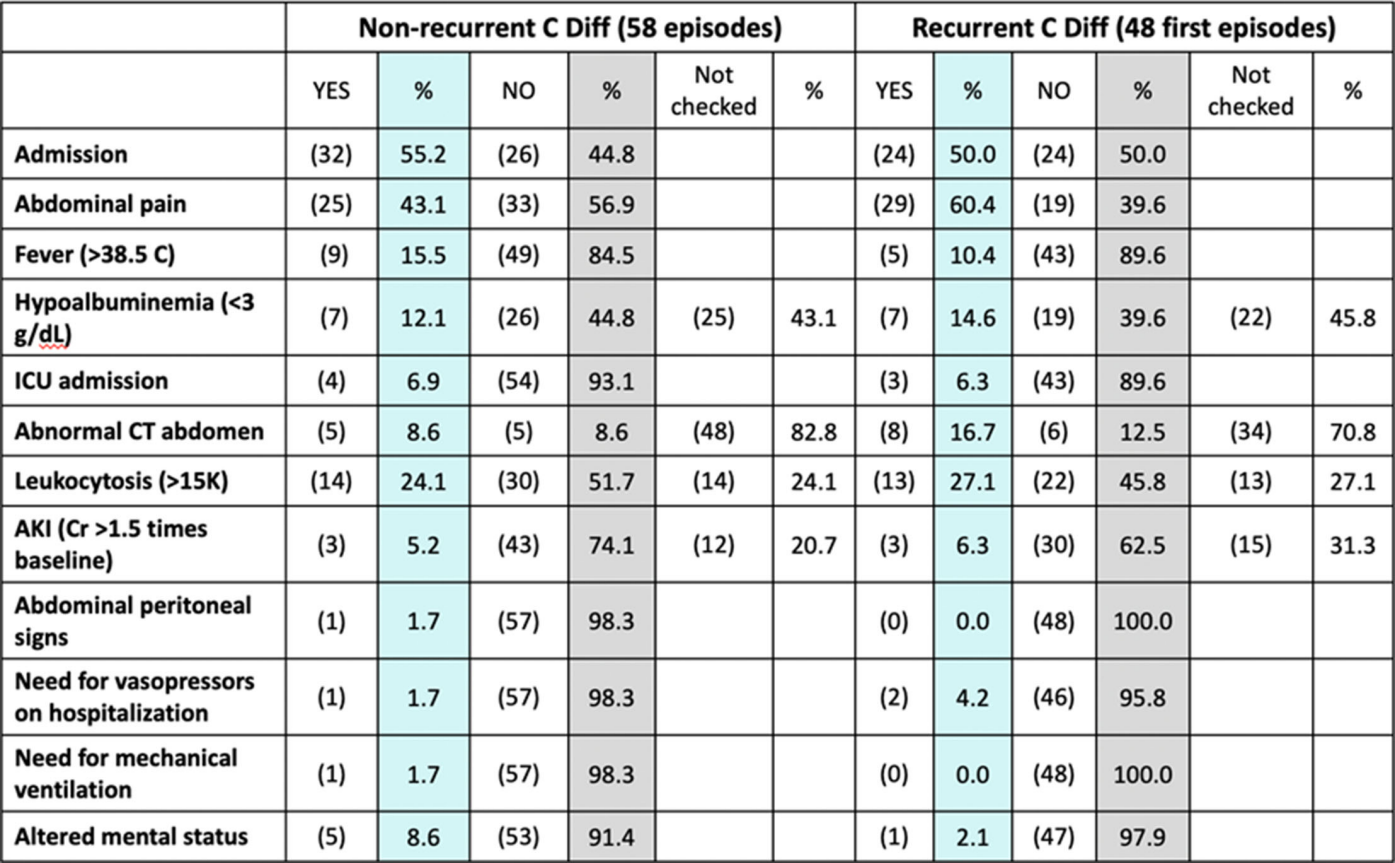# Clinical Characteristics and Fecal Microbiome in Recurrent Versus Nonrecurrent *Clostridioides difficile* Infection

**DOI:** 10.1017/ash.2021.77

**Published:** 2021-07-29

**Authors:** Swapnil Lanjewar, Ashley Kates, Lauren Watson, Nasia Safdar

## Abstract

**Background:** Up to 30% of patients with *Clostridioides difficile* infection (CDI) develop recurrent infection, which is associated with a 33% increased risk of mortality at 180 days. The gut microbiome plays a key role in initial and recurrent episodes of CDI. We examined the clinical characteristics and gut microbial diversity in patients with recurrent (rCDI) versus nonrecurrent CDI at a tertiary-care academic medical center. **Methods:** Stool samples were collected from 113 patients diagnosed with CDI between 2018 and 2019. Clinical and demographic data were extracted from the electronic medical record (Table 1), and 16S rRNA sequencing of the v4 region was carried out on the Illumina MiSeq using 2×250 paired-end reads. Sequences were binned into operational taxonomic units (OTUs) using mothur and were classified to the genus level whenever possible using the ribosomal database project data set version 16. Alpha diversity was calculated using the Shannon diversity index. Β diversity was calculated using the Bray-Curtis dissimilarity matrix. Differential abundance testing was done using DESeq to assess taxonomic differences between groups. A *P* value of .05 was used to assess significance. **Results:** In total, 55 patients had rCDI (prior positive *C. difficile* polymerase chain reaction in last 7–365 days) and 58 had nonrecurrent CDI (Table 1). Patients with rCDI had a higher frequency of organ transplant and comorbidity. No differences in α not β diversity were observed between groups. Also, 4 OTUs were more abundant in those with rCDI: *Ruminococcus* (n = 2), *Odoribacter*, and *Lactobacillus*. Patients with rCDI had microbiomes with greater proportions of Bacteroidetes (27% of OTUs) compared to the nonrecurrent group (18%) as well as fewer OTUs belonging to the *Firmicutes phyla* compared to the nonrecurrent patients (56% vs 59%). Among the rCDI patients, those experiencing 2 or more recurrences had greater abundances of Bacteroides and *Ruminococcus*, while those experiencing only 1 recurrence had significantly greater abundances of *Akkermensia*, *Ruminococcus*, *Streptococcus*, *Roseburia*, *Clostridium* IV, and *Collinsella* compared to those with only 1 recurrence (Table 2). **Conclusions:** Patients with rCDI had a more impaired microbiome than those with initial CDI. *Ruminococcus* OTUs have been previously indicated as a risk factor for recurrence and treatment failure, and they were significantly more abundant in those with rCDI and among those with multiple recurrences. The greatest differences in the microbiome were observed between those with 1 recurrence compared to those with multiple recurrences. Interventions for gut microbiome restoration should focus particularly on those with recurrent CDI.

**Funding:** No

**Disclosures:** None

**Table 1.**
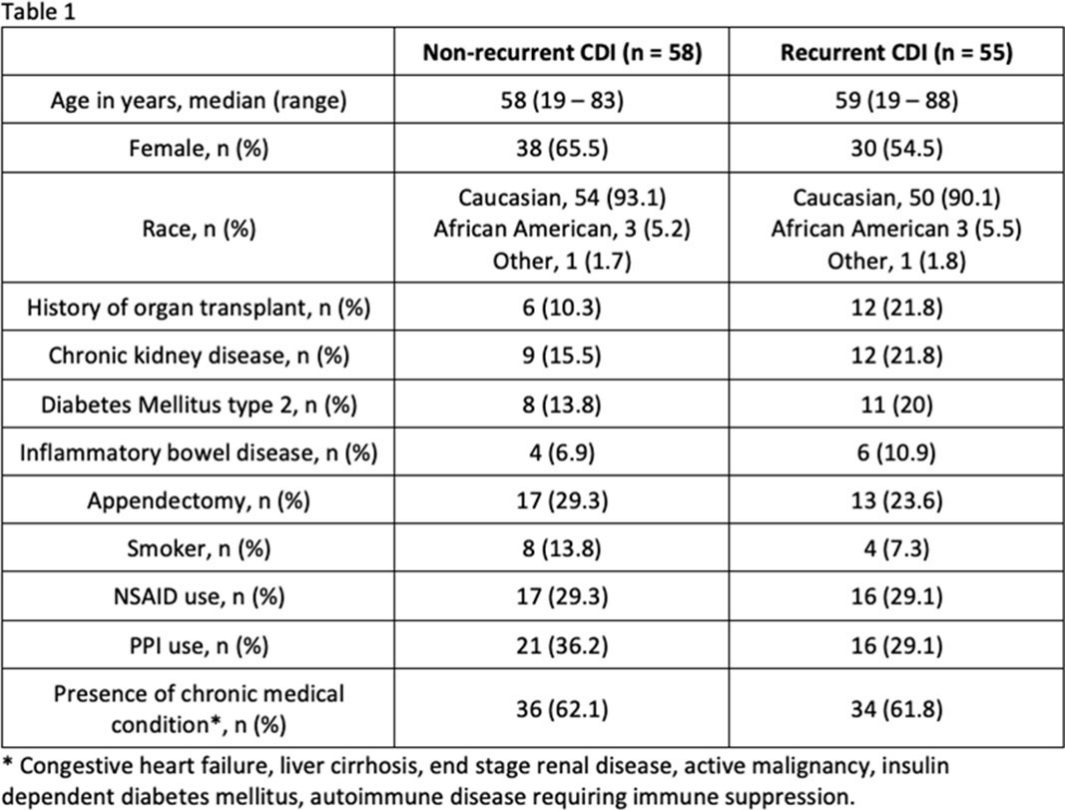

**Table 2.**